# Development and In Vitro/In Vivo Evaluation of pH-Sensitive Polymeric Nanoparticles Loaded Hydrogel for the Management of Psoriasis

**DOI:** 10.3390/nano11123433

**Published:** 2021-12-17

**Authors:** Muhammad Imran Asad, Dildar Khan, Asim ur Rehman, Abdelhamid Elaissari, Naveed Ahmed

**Affiliations:** 1 Department of Pharmacy, Quaid-i-Azam University, Islamabad 45320, Pakistan; miasad@bs.qau.edu.pk (M.I.A.); dildarafridi3@gmail.com (D.K.); arehman@qau.edu.pk (A.u.R.); 2 CNRS, University Claude Bernard Lyon–1, ISA-UMR 5280, 69622 Lyon, France; elaissari@lagep.univ-lyon1.fr

**Keywords:** psoriasis, methotrexate, Eudragit E100, nanoparticles, chitosan, hydrogel, in vitro release, imiquimod induced psoriasis model

## Abstract

Methotrexate (MTX), the gold standard against psoriasis, poses severe problems when administered systemically viz increased toxicity, poor solubility and adverse reactions. Hence, a topical formulation of MTX for the management of psoriasis can be an effective approach. The present study aimed to develop an MTX based nanoparticle-loaded chitosan hydrogel for evaluating its potential efficacy in an imiquimod-induced psoriatic mice model. MTX-NPs loaded hydrogel was prepared and optimized using the o/w emulsion solvent evaporation method. Particle size, zeta potential, entrapment efficiency, in vitro drug release, ex vivo permeation, skin irritation and deposition studies were performed. Psoriatic Area and Severity Index (PASI) score/histopathological examinations were conducted to check the antipsoriatic potential of MTX-NPs loaded hydrogel using an imiquimod (IMQ)-induced psoriatic model. Optimized MTX-NPs showed a particle size of 256.4 ± 2.17 nm and encapsulation efficiency of 86 ± 0.03%. MTX-NPs loaded hydrogel displayed a 73 ± 1.21% sustained drug release in 48 h. Ex vivo permeation study showed only 19.95 ± 1.04 µg/cm^2^ of drug permeated though skin in 24 h, while epidermis retained 81.33% of the drug. A significant decrease in PASI score with improvement to normalcy of mice skin was observed. The developed MTX-NPs hydrogel displayed negligible signs of mild hyperkeratosis and parakeratosis, while histopathological studies showed healing signs of mice skin. So, the MTX-NPs loaded hydrogel can be a promising delivery system against psoriasis.

## 1. Introduction

Psoriasis, an inflammatory disease of the skin with completely unknown cause, is a T-cell mediated autoimmune chronic disorder of hyper-activated keratinocytes [1]. The overexpressed proinflammatory cytokines and chemokines produced by Th1 cells make this disease a multifactorial disorder. These Th1 cells are primarily responsible for the production of tumor necrotic factor alpha (TNF-α) and cytokines, i.e., interleukins (IL)–6, IL- 17, IL–22 and IL–23 [2]. These cytokines and chemokines together result in auto-amplification and hyper-proliferation of the epidermis, along with cutaneous inflammation and dermal neovascularization, leading to the formation of plaque psoriasis [3]. Dry, scaly and inflamed patches on elbows, knees and scalp differentiate it; hence, the diagnosis of psoriasis depends upon the health condition and appearance of the skin. Genetic factors are mainly responsible for the emergence of this disease as it is noncontagious. Therapy for this disease depends on the severity of skin condition, which can range from small to local patches. Topical and systemic medications for psoriasis are available on the market for symptomatic management. However, in the local delivery of drugs, the skin poses a restriction [4]. To overcome this restriction and make sure that maximum drug reaches the deepest layers of the skin, novel techniques have been developed to boost drug delivery within safety ranges [5]. Conventional therapy for psoriasis normally includes steroidal creams, ultraviolet radiations and immune system suppressors. The major problems related to conventional therapies are extensive drug distribution, insoluble drug moiety in formulations and metabolism [6]. However, nanocarriers have advantages over conventional therapies with reduced dosage, efficient targeting and an improved safety profile [7].

Nanotechnology-centered drug delivery has become an attractive and captivating part of pharmaceutical science for encapsulating and delivering therapeutic drugs more specifically to the tissues [8]. The precise delivery of therapeutic agents to the target site is a demanding area in material science. Smart delivery techniques have been used recently using polymeric nanomaterials and hydrogels with ease in synthesis and cost effectiveness [9]. Nanotechnology has been functionally applied to topically treat immune-related skin diseases. Topical treatment due to low systemic toxicity and accurate targeting ability is recommended in about 90% of mild to moderate psoriatic patients [10]. Polymeric nanoparticles have been synthesized to reduce toxicity, boost therapeutic efficacy and enhance drug penetration due to good compatibility with lipophilic drugs. They also have a high affinity towards skin barriers and has no toxicity, as seen in the case of systemically administered drugs [11]. Topical therapy is commonly used to mitigate the symptoms in most of psoriatic patients [12].

Polymers with natural and synthetic properties are used as drug carriers to improve delivery because they degrade into nontoxic materials in various pH environments [13]. Eudragit E100 (E100), being a cationic copolymer, has dimethyl aminoethyl methacrylate (DMAEA), butyl methacrylate/methyl methacrylate in a 2:1:1 ratio. This polymer is most commonly used in floating drug delivery systems, microparticles, ophthalmic solutions, nanoparticles, transdermal sprays and films, etc., [14]. The Eudragit series of polymers have high drug loading capacities and are well tolerated by the skin, especially, E100, which possesses the adhesive properties suitable for the controlled release of drugs from the matrix system. These polymers also own the defined permeability for dissolved drugs and have a swelling capacity for water [15].

Methotrexate (MTX), an antifolate drug, is widely used for its immunomodulatory and antiproliferative effects in the field of dermatology. MTX is weak dicarboxylic acid with a molecular mass of 454.5 g/mol. It has poor aqueous solubility (0.01 mg/mL) with low permeability (ClogP = 0.53) and pKa values of 4.7–5.5 [16]. MTX is administered systemically for several dermatologic disorders, including atopic dermatitis, psoriasis, alopecia areata, mycosis fungoides, dermatomyositis, sarcoidosis, Crohn’s disease, cutaneous lupus erythematosus and multiple sclerosis. The side effects of systemic MTX include hepatic dysregulations, gastrointestinal disorders, pneumonitis, nephrotoxicity, hematologic disorders and infections [17].

Although MTX is effectively used to treat autoimmune and inflammatory diseases, it has some limitations, such as low bioavailability due to low permeability and poor water solubility [18,19]. Furthermore, it is eliminated through the kidneys within a very short period of time, due to which it has a short half-life and, hence, a lower concentration of drug is available at the target tissues [20].

The foremost objective of the present research was to formulate Eudragit E100 nanoparticles for topical delivery of MTX to manage psoriasis. E100 is extensively utilized in oral and topical preparations and mostly considered non-irritant, nontoxic and effectively safe for human beings [21]. In addition, the positive charge of particles of amino groups present in E100 enhances the cellular uptake of biomolecules and protects them from enzymatic attack by proton sponge effect, providing storable nanoparticulate without cryoprotectant after freeze-drying [22]. Methotrexate nanoparticles (MTX-NPs) once prepared were embedded into chitosan hydrogel. The hydrogel serves as a carrier system for the incorporated drug to be delivered at the specific site with protection of infected skin by maintaining the moisture content [23,24]. Chitosan, a cationic polymer is usually used in sustained drug delivery systems to prolong the release of the drug [25,26]. Chitosan-based hydrogels show biological properties such as antimicrobial activity against many bacteria [27], bioadhesivity, biodegradability, biocompatibility and non-toxicity. It has the ability to provide a matrix for therapeutic agents to be delivered locally with better wound-healing and bacteriostatic activities [28,29]. The prepared nanoformulation was subjected to various characterizations to determine entrapment efficiency (EE), distribution of particle size, zeta potential, permeation and skin irritation studies, drug release profile, rheology, morphology, bioadhesive properties and in vivo studies. Hence, a novel pH sensitive formulation of Methotrexate has been formulated and loaded in the hydrogel with promising results.

## 2. Materials and Methods

### 2.1. Materials

Eudragit^®^ E100 (E100) molecular weight (47,000 g/mol) Evonik Röhm GmbH (Darmstadt, Germany), ethanol ≥ 99.8%, dimethyl sulfoxide (DMSO), polyvinyl alcohol (PVA), triethanolamine, monobasic potassium phosphate, glutaraldehyde (GA), chitosan (degree of deacetylation 94%), and potassium dihydrogen phosphate were purchased from Sigma-Aldrich (Darmstadt, Germany). Glacial acetic acid, hydrochloric acid (HCl) and sodium hydroxide (NaOH) were purchased from Merck (Darmstadt, Germany). Synthetic membrane obtained from Fluka (Darmstadt, Germany), methotrexate (MTX) was gifted by Pfizer Pakistan Limited Islamabad.

### 2.2. Methods

#### 2.2.1. Optimization of MTX Loaded NPs

Design-Expert^®^ Stat-Ease software (version 10.0) was applied to optimize NPs. The amount of polymers (E100), drugs (MTX) and surfactants (PVA) were under study, whereas the aqueous to organic phase ratio remained constant. Using Box–Behnken Design, the software generated 15 different runs. Each run was carried out and two responses, particle size and encapsulation efficiency, were calculated.

#### 2.2.2. Formulation of Methotrexate Loaded NPs

MTX-NPs were synthesized using the o/w emulsion solvent evaporation technique with minor modifications [30]. MTX (3–6 mg) was dissolved in 0.5 mL of DMSO, and Eudragit E100 (25–200 mg) was solubilized in 4 mL of ethanol. Drug and polymer solutions were combined for the organic phase. Then, the prepared organic phase was injected dropwise into the aqueous phase, comprising PVA (25–170 mg) in 15 mL distilled water under high speed homogenization (5000–25,000 rpm) for a specific period of time, i.e., 0.5–6 min. The prepared solution was then subjected to vacuum rotary evaporator for evaporation of organic solvents. After evaporation, the formulation was centrifuged at 13,500× *g* rpm (22,413 RFC) for 30 min at 4 °C to collect the pellets [31]. The prepared nanoparticles were then washed with DMSO to remove the adherent free drug on the NPs’ surface. Furthermore, the formulation was freeze-dried at −60 °C for 6 h for further characterization [32]. The freeze-dried MTX-NPs were kept in airtight glass vials. Blank NPs were prepared using the same method mentioned above but without the addition of the drug.

### 2.3. Characterization of Prepared MTX-NPs

#### 2.3.1. Particle Size, Zeta Potential and Polydispersity Index (PDI)

Particle size and PDI of NPs’ dispersion were determined through Zeta Sizer (Malvern Instruments, Worcestershire, UK). Each sample was diluted 10 times with double distilled water, vortexed and placed in cuvettes at 90° of scattering between a scanning size range of 50–3000 nm, by maintaining temperature at 25 °C. Triplicate measurements were taken for each sample [33].

Zeta potential of prepared NPs was found using Malvern Zeta Sizer (Malvern, London, UK). The NPs after freeze drying were redispersed in distilled water and placed in neat and clear disposable cuvettes for the measurement of zeta potential by running the samples at 100 zeta runs at 25 °C [34].

#### 2.3.2. Encapsulation Efficiency (EE)/Drug Loading (DL)

To calculate encapsulation efficiency, the MTX-NPs formulation was centrifuged for 45 min at 4 °C at 13,500× *g* rpm. As free MTX was suspended in supernatant, we diluted it with DMSO (dimethyl sulfoxide) which dissolved MTX, but E100 NPs were not soluble in it. The absorbance was taken using a UV-visible spectrophotometer at 303 nm [35]. EE and DL were estimated using Equations (1) and (2), respectively [36].

(1) $$\%\;(\mathrm{EE})=\frac{\mathrm{Total}\;\mathrm{MTX}\;\mathrm{added}\;\mathrm{in}\;\mathrm{formulation}-\mathrm{MTX}\;\mathrm{present}\;\mathrm{in}\;\mathrm{the}\;\mathrm{supernatant}}{\mathrm{Total}\;\mathrm{MTX}\;\mathrm{added}\;\mathrm{in}\;\mathrm{formulation}}\;\times \;100$$

Similarly, the dug loading was calculated by equation given below

(2) $$\%\;(\mathrm{DL})=\frac{\mathrm{Total}\;\mathrm{MTX}\;\mathrm{added}\;\mathrm{in}\;\mathrm{formulation}-\mathrm{MTX}\;\mathrm{present}\;\mathrm{in}\;\mathrm{the}\;\mathrm{supernatant}}{\mathrm{weight}\;\mathrm{of}\;\mathrm{polymer}+\mathrm{Total}\;\mathrm{MTX}\;\mathrm{added}\;\mathrm{in}\;\mathrm{formulation}}\;\times \;100$$

#### 2.3.3. Fourier Transform Infrared Spectroscopy (FT-IR)

FTIR analysis was performed to check any possible interaction between various constituents of MTX-NPs. Samples of E100, MTX, PVA, freeze dried blank and MTX-NPs were prepared and studied through FTIR spectrometer (Perkin Elmer, Waltham, MA, USA).

#### 2.3.4. X-ray Diffraction (XRD) Analysis

XRD analysis of E100, MTX, PVA, blank and MTX-NPs were carried out using an X-ray diffractometer (Theta, Stoe-Stadi, Darmstadt, Germany) to check the crystalline and amorphous form of individual constituents and the formulation. The scanning range of 2 θ was from 5° to 90° [16].

#### 2.3.5. Differential Scanning Calorimetry (DSC)

DSC thermograms of MTX, E100 and MTX-NPs were obtained using DSC (Mettler Toledo, Schwerzenbach, Switzerland). Samples were placed in a heating range of 25 –200 °C with a rate of 10°C/min. Samples weighing 2 mg were placed in an aluminum pan for analysis with an empty reference pan [37].

#### 2.3.6. Scanning Electron Microscopic Analysis

Morphology of MTX-NPs was observed under scanning electron microscope (JSM–6360LV Scanning Microscope; Jeol, Tokyo, Japan). A dispersion of NPs was dried on an aluminum stub overnight under vacuum and gold sputtering coating was carried out using a gold sputter module with high-vacuum evaporator (JFC–1100 fine coat ion sputter; Jeol, Tokyo, Japan). Photomicrographs of coated samples were taken after scanning at an accelerated voltage of 15 kV [30].

### 2.4. Preparation of Chitosan Hydrogel

Chitosan hydrogels were formulated by solubilizing 2% (*w*/*v*) chitosan (medium molecular weight) in 1% (*v*/*v*) aqueous acetic acid on a hot plate. When the chitosan was completely dissolved, crosslinker glutaraldehyde (200–600 µL) was added to the solution by stirring on a hot plate. Finally, a few drops (200 µL) of triethanolamine (gelling agent), which helps in gel formation and increases stability of gel [38], were added to the solution till a hydrogel-like consistency was obtained. Triethanolamine is used in formulations or products intended to be applied on skin such as cosmetics, as a surfactant, or pH adjuster [39]. For MTX-NPs loaded hydrogel, MTX-NPs were added to the chitosan solution with continuous stirring before the addition of the cross-linker.

#### Characterization of Hydrogel

##### Physical Examination

The synthesized MTX-NPs loaded hydrogel was visually examined for its appearance, color and consistency [12].

##### Viscosity Measurement

The viscosity of MTX-NPs loaded hydrogel was determined using the Brookfield viscometer (DV-I Prime, Middleboro, MA, USA). For viscosity measurement, 20 g of hydrogel was taken in a beaker and Spindle no. S63 was dipped in it at 0.5, 1, 2.5, 10, 20, 50 and 100 rpm at room temperature [40].

##### Spreadability Measurement

MTX-NPs loaded hydrogel was evaluated for spreadability by placing 0.5 g hydrogel in 20 × 20 acrylic plates within a circle of 1 cm diameter. A 500 g weight was placed on hydrogel for 1 min, which resulted in the spreading of hydrogel. A linear scale was used to measure the extension in the diameter. Equation (3) was used to evaluate the results of spreading area with respect to applied mass [37].

(3) $$\mathrm{Si}=d^{2}\;\times \;\frac{\pi }{4}$$

Si stands for spreadability

d = diameter of the circle

##### Extrudability and pH Measurement

A collapsible tube containing 20 g of hydrogel was compressed at the crimped end by applying a constant load of 1kg to determine the extrudability. When the cap was detached, the hydrogel extruded until the pressure dissipated. The hydrogel extruded out was collected and weighed [41].

The pH of hydrogel was determined using a digital pH meter (calibrated). MTX-NPs loaded hydrogel (2 g) was dispersed in 25 mL of double distilled water and electrode of pH meter was placed in it and readings were noted. This experiment was carried out in triplicate.

##### Drug Content of Hydrogel

To quantify the drug content of hydrogel, 1 g of hydrogel was mixed in 10 mL of phosphate-buffered solution (PBS) at a pH of 5, with constant stirring on a hot plate. This mixture of hydrogel was filtered and examined at 303 nm wavelength on a double beam UV-Vis spectrophotometer, keeping PBS as reference. The drug content was estimated through Equation (4) [42] which defines the estimation of drugs in the hydrogel

(4) $$\%\;\mathrm{drug}\;\mathrm{content}=\frac{\mathrm{Actual}\;\mathrm{amount}\;\mathrm{of}\;\mathrm{drug}\;\mathrm{detected}}{\mathrm{Amount}\;\mathrm{of}\;\mathrm{drug}\;\mathrm{in}\;\mathrm{formulation}}\times 100$$

### 2.5. In Vitro Drug Release from NPs and NPs Loaded Hydrogel

In vitro drug release studies for free MTX as control formulation, MTX-NPs and MTX-NPs loaded hydrogel were conducted at various pH values (5 and 7.4) using the dialysis method [43]. For this purpose, 5 mL of MTX dispersion, MTX-NPs dispersion and 5 g hydrogel were placed in dialysis membranes (12 kDa molecular weight, Sigma-Aldrich, St. Louis, MO, USA) separately. Each dialysis membrane was placed in a beaker with 50 mL of PBS solution. Then, the beaker was placed in a water bath shaker (Memmert water bath, WNB 14) maintained at 37 ± 0.5 °C with constant shaking at a rate of 50 rpm. The samples (2 mL) at predetermined intervals of time, i.e., 0.5, 1, 2, 4, 6, 8, 12, 24, 48 and 72 h were collected and replaced with an equivalent volume of freshly prepared PBS solution to maintain the sink conditions. The MTX was estimated through UV-Vis spectrophotometer at 303 nm. Different kinetic models, i.e., zero order, first order, Higuchi, Korsmeyer-Peppas and Hixson-Crowell models were employed on the release data using DD solver software to anticipate the drug release mechanism and diffusion characteristics of MTX from the MTX-NPs and MTX-NPs loaded hydrogel [42]. The slopes of respective plots estimated the release constant. The best-fit model was the one with an R^2^ value near to 1.

### 2.6. Cell Viability Assay on Blood Lymphocytes

Lymphocytes of human blood were isolated using the formerly defined protocol [44]. A 3 mL blood sample taken from a healthy donor was diluted (1:1) with PBS. Then, it was covered with almost 2 mL of Histopaque–1077 with centrifugation at 800× *g* for 20 min. To collect the pellets of lymphocytes, the buffy coat was obtained into PBS (5 mL) and again centrifuged at 350× *g* for 4 min. The obtained pellet was placed in 1 mL of RPMI–1640.

Methylthiazole tetrazolium (MTT) assay was carried out to verify the cytotoxic effects of free MTX and MTX-NPs loaded hydrogel in lymphocytes, following the previously described protocol [45]. Firstly, lymphocytes were placed in each well and a CO_2_ incubator was used for the attachment of lymphocytes. After incubation, cells were exposed to different concentrations, i.e., 0 to 100 μg/mL of free MTX and MTX-NPs loaded hydrogel for 24 h. Then, reagent 1 (10 μL) (methyl tetrazolium, 1%) was added in each well and incubated for 4 h in CO_2_ incubator until development of purple formazan crystals. Then, 100 μL of reagent 2 (DMSO and 10% SDS with 0.01 N HCl) was added to the plate and incubated for 12 h in CO_2_ incubator, followed by absorbance measurement at 590 nm employing microplate reader. Triplicate measurements were carried out for this experiment.

### 2.7. Ex Vivo Skin Permeation Studies

#### 2.7.1. Preparation of Skin

The healthy mice skin used for the permeation studies was obtained from a pharmacology laboratory in the Department of Pharmacy, Quaid-i-Azam University, Islamabad (Pakistan). Abdomen skin without fats was used after removing all hair with a razor. The integrity of skin was visually inspected and it was kept in PBS (pH 5) for half an hour [46].

#### 2.7.2. Ex Vivo Skin Permeation

Franz diffusion cell was used to carry out the ex vivo permeation studies. The skin with an area of 0.77 cm^2^ was placed on the donor compartment. The donor compartment contained free MTX hydrogel, marketed cream, dispersion of MTX-NPs and MTX-NPs loaded hydrogel, while the receptor compartment was filled with PBS (pH 7.4) with a temperature maintained at 37 ± 0.5 °C. Samples (each 1 mL) were collected from the receptor compartment at predetermined time intervals, i.e., 0.5, 1, 2, 3, 4, 6, 8, 12 and 24 h, with immediate replacement of diffusion medium. A UV-Vis spectrophotometer was used to analyze the samples at 303 nm wavelength. Permeation studies in triplicate were carried out to quantify permeated drug per unit area and time. These results were plotted on MS-Excel to obtain the results. Equation (5) was used to measure the flux [47].

Jss = dQ/dtA(5)

where Jss = Flux or permeation at steady state (μg/cm^2^/h), A = Area of skin tissue (cm^2^) through which drug diffusion occurs, (dQ/dt) = Quantity of drug (μg/h) passing across skin per unit time at a steady state.

### 2.8. Skin Deposition Study

The total quantity of the drug held in the layers of skin was estimated using the tape stripping method [48]. At the end of the permeation studies, i.e., at 24 h, the excess amount of drug on the surface of the skin was cleaned with a buffer solution. Then, ScotchTM tape (19 mm, 3M, Saint Paul, MN, USA) was utilized using the tape stripping method. The initial strips with the drug were thrown away as they could have parts of the drug that remained on the surface of the skin. Then, stratum corneum was detached entirely by tape stripping for 15 times. Remaining skin samples and stripped tapes were soaked overnight in PBS (pH 5) with 5% methanol and 1% *w*/*v* albumin. Onwards, sonication was performed for 1 h to extract the drug. The samples after extraction were then analyzed using a UV-Vis spectrophotometer.

### 2.9. Skin Irritation Studies

The potential of prepared formulations and marketed cream was evaluated by conducting a skin irritation test (Draize patch test) on mice with slight modifications. The hair on the back of mice were removed 24 h prior to the study by anesthetizing them. Animals with good health were distributed into five groups. For group 1 (control group) no formulation was used, group 2 (negative control) was treated with 0.8% formalin solution, group 3 was treated with free MTX loaded hydrogel (prepared by dispersing MTX in hydrogel), group 4 was treated with marketed tacrolimus cream, whereas group 5 was treated with MTX-NPs loaded hydrogel. A single dose (0.5 g) of formulation was applied uniformly on the shaved skin (2 cm^2^) of mice. Afterwards, the skin was washed using saline water to check any noticeable changes such as edema and erythema at 24, 48 and 72 h. The mean score was determined depending on the degree of erythema and edema [49].

### 2.10. In Vivo Antipsoriatic Activity

To evaluate the potency of developed MTX-NPs loaded hydrogel for antipsoriatic activity, an Imiquimod (IMQ) induced psoriatic plaque model was used [50]. After approval from the Bio-Ethical Committee (BEC) Quaid-i-Azam University Islamabad, Pakistan under reference no. (#BEC-FBS-QAU2020–260), antipsoriatic studies were performed. Male BALB/c mice of 6–8 weeks old, with a weight ranging 20–30 g, were utilized to conduct antipsoriatic activity. Mice were kept under standard laboratory environments of temperature and humidity, i.e., 25 ± 2 °C and 55 ± 55% RH with unrestricted availability of food and water. The dorsal backside of mice was shaved using a Sterling–2 hair clipper prior to the study. The mice were divided into five groups. Psoriasis or hyperplasia was induced, except in group 1, by topically applying marketed imiquimod (IMQ) cream (Aldara^TM^ 5% cream *w*/*w*, (MEDA AB, Pharma) on the dorsal side’s shaved skin and right ear of healthy mice with an amount equal to 62.5 mg for successive 7 days [51]. From day 8, the group 3, 4 and 5 were treated with free MTX hydrogel, marketed cream and MTX-NPs loaded hydrogel, respectively. The severity of inflammation was measured by psoriasis area severity index (PASI) [1], to check parameters such as erythema, scaling and ear thickness. The treatment continued for the 10 consecutive days after psoriasis induction. The intensity of psoriatic symptoms such as thickness, redness and scaling were assessed again as none (0), mild (1), moderate (2), severe (3) and very severe (4) and compared with scores before treatment. These results were drawn by taking time on *x*-axis and PASI score (0–4) along *y*-axis.

### 2.11. Histopathological Studies

Histopathological studies were conducted to assess the pathological variations that occurred during the development of the psoriatic model. The skin samples gathered at the end of the study were fixed with 10% formalin and subjected to histopathological examination. The collected skin tissues were then fixed in paraffin for partitioning, followed by immediate hematoxylin and eosin (H&E) staining and mounting on slides, following the standard technique. The developed slides were checked under an inverted microscope (Axiovert A1, Carl Zeiss, Oberkochen, Germany) for typical pathologic features such as thickness of stratum corneum (hyperkeratosis), nuclei retention in the stratum corneum (parakeratosis), epidermis proliferation (acanthosis) and inflammatory infiltrate [52].

### 2.12. Spleen to Body Weight (Spleen/Body Weight Percent)

In the human immune system, the spleen is the largest organ with respect to body weight [53] and is considered a very sensitive indicator of depletion or stimulation. The increase in spleen/body weight percent is the indication of higher number of spleen cells, which suggests the progression of disease due to activation of the immune system [11]. At the end of experiment, spleens from the mice were harvested and spleen/body weight percent was measured.

### 2.13. Enzyme-Linked Immunosorbent Assay

Psoriasis is distinguished by varied levels of key inflammatory cytokines such as IL–1β, IL–6, IL–17, IL- 22 and TNF-α [54]. The skin samples treated with various prepared formulations were collected at the end of the efficacy study and stored at −80 °C until further use. Serum levels of TNF-α, IL–6, IL–17 and IL–22 were measured using the commercial enzyme-linked immunosorbent assay (ELISA kits) [Invitrogen] in accordance to the manufacturer’s instructions.

### 2.14. Stability Studies

MTX-NPs and MTX-NPs loaded hydrogel were subjected to three different temperatures for a period of 6 months. The samples were sealed in glass vials and subjected to three varying temperatures, i.e., 4 ± 2 °C, 25 ± 52 °C and 40 ± 2 °C/75% RH and sampling was carried out at the end of each week to determine entrapment efficiency, pH and percent drug content [55].

### 2.15. Statistical Analysis

The degree of statistical significance was established by assessment of variance (ANOVA), observed by Bonferroni’s test for various comparisons, with the use of GraphPad Prism (Version 5.01) software. Significance was supposed at *p* < 0.05 (level of probability).

## 3. Results

### 3.1. Optimization of Nanoparticles

Fifteen (15) formulations generated by Design Expert were prepared with various concentrations of polymer (E100), drug (MTX) and surfactant (PVA). Particle size, zeta potential and encapsulation efficiency of formulations are displayed in Table 1. Formulation 3 shows the required particle size and highest EE.

#### 3.1.1. Impact of PVA, E100 and MTX Concentrations on Encapsulation Efficiency of NPs

**Figure 1:**
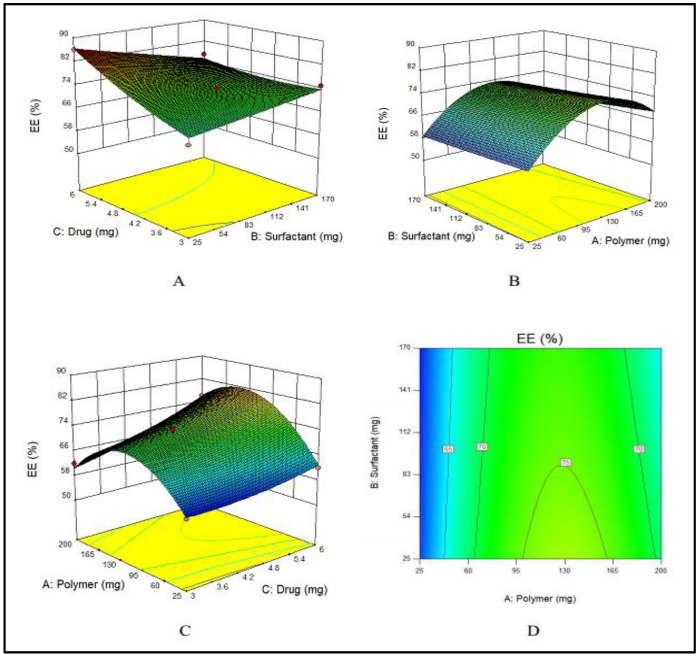
(**A**–**C**) showing the 3D surface plot for impact of PVA (surfactant), E100 (polymer) and MTX (drug) on EE, respectively while (**D**) showing the combined effect of PVA, E100 and MTX on EE.

PVA has an indirect effect on the EE, i.e., with an increase in the amount of PVA, EE decreases, as displayed in Figure 1A. This effect was ascribed to the fact that with an increased amount of PVA, the drug leaked into the external phase due to cavitation in the external phase that lowered the EE. E100 has a direct influence on the EE. By increasing the quantity of E100 from 25 to 140 mg, EE was increased. Meanwhile, a further increase in the amount of polymer decreased the EE as shown in Figure 1B. Similarly, by increasing the amount of drug, the EE was increased (Figure 1C). These combined effects are depicted in Figure 1D.

The F-value (11.70) of the model suggests its significance. There is only a chance of 0.73% that an F-value could be large because of noise. The values of “Prob > F” less than 0.0500 suggested that model terms are significant. A, C and BC are significant model terms. The values larger than 0.1000 showed that the model terms are not significant.

#### 3.1.2. Impact of PVA, E100 and MTX Concentrations on Particle Size of NPs

**Figure 2:**
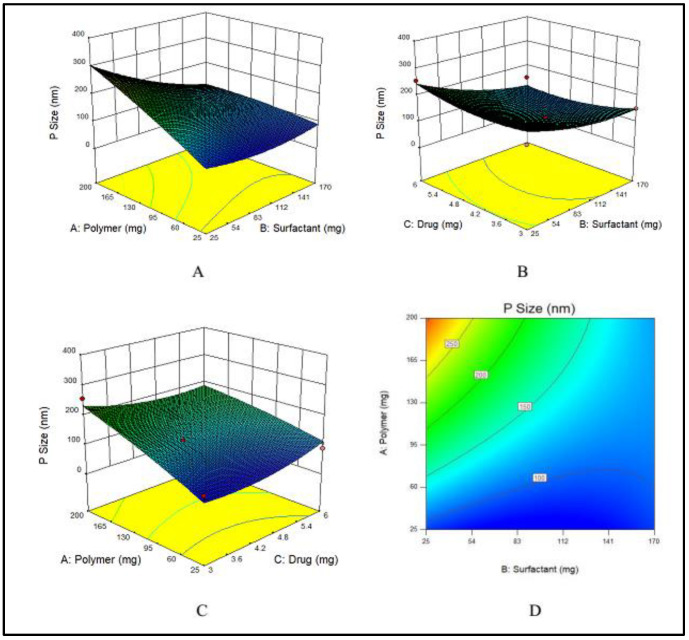
(**A**–**C**) showing 3D surface plot for impact of PVA (surfactant), E100 (Polymer) and MTX (drug) on particle size of MTX-NPs, respectively while (**D**) showing the combined effects of PVA, E100 and on particle size.

Nanoparticles are usually stabilized by commonly used emulsifiers such as PVA [56]. The hydroxyl groups present in PVA cause hydration at the surface of nanoparticles that stabilize them [16]. To evaluate the effects of PVA on particle size, various concentrations of PVA varying from 25 to 170 mg were used. Particle size has a prime importance as it directly effects the drug release and stability of the prepared formulation [57]. The particle size, a dependent variable, indicated a wide-ranging variations from 75 to 323 nm at various concentrations of the two independent variables, i.e., E100 and PVA. The maximum particle size (323 nm) was obtained at the lowest concentration of PVA, while by increasing the PVA concentration, the particle size decreased as shown in Figure 2A.

An increase in the concentration of the polymer had a direct effect on particle size, which increased when the concentration of EE100 was increased (Figure 2B).

The drug concentration has no major effect on the particle size of NPs, however at the lowest concentration of drug, the particle size was maximum, and by increasing the concentration, the size gradually decreased, but again at maximum concentration of drug, the particle size was higher. The effect of the drug on particle size is demonstrated in Figure 2C. The combined effects of the polymer and surfactant on the particle size is demonstrated in Figure 2D.

The Model F-value of 5.49 indicates that the model is significant. Therefore, due to noise, there are only 3.76% chances that an F-value could be large. Values of “Prob > F” less than 0.0500 suggest that the model terms fall in the significant range. In the case of A, B, AB, model terms are significant. The values larger than 0.1000 suggest that model terms show non-significant results.

### 3.2. Physico-Chemical Characterization of MTX-NPs

#### Particle Size, Zeta Potential and Polydispersity Index

**Figure 3:**
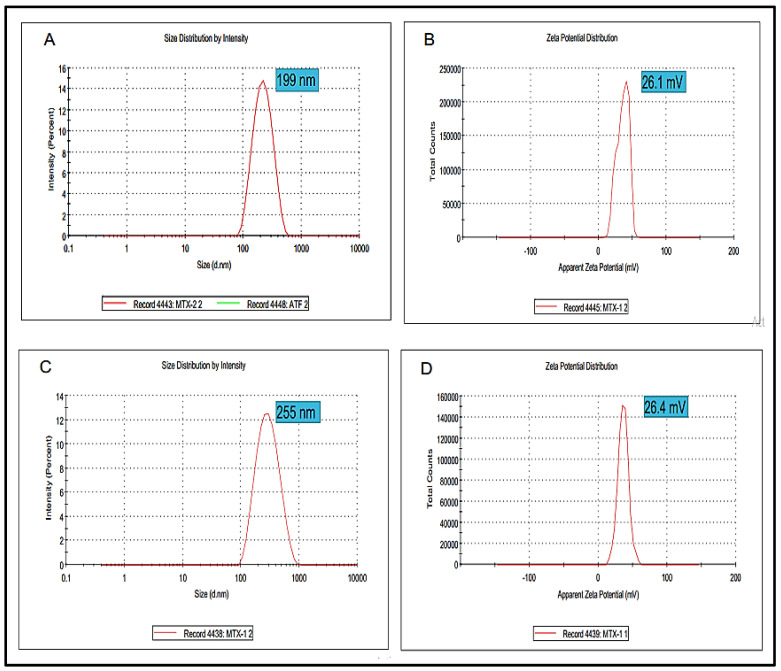
(**A**,**B**) showing particle size, zeta potential and PDI of blank NPs while (**C**,**D**) showing particle, zeta potential and PDI of MTX-NPs.

Particle size is one of the extremely important factors that directly influences the drug release and stability of the prepared formulation. The Blanks NPs exhibited a particle size of 199 nm with a zeta potential value of 26.1 mV (Figure 3A,B). Wherein, the optimized MTX-NPs had a particle size of 255 nm with zeta potential of 26.4 mV (Figure 3C,D). Higher positive values suggested good zeta potential, which in turn showed the stability of NPs [58]. PDI for blank and MTX-NPs was 0.21 and 0.17, respectively.

### 3.3. Encapsulation Efficiency (EE)/Drug Loading (DL)

EE is the percentage of active constituent or drug successfully entrapped or encapsulated into the NPs, while drug loading is the amount of drug loaded per unit weight of the NPs. MTX-loaded NPs with various amounts of drug were statistically significant (*p* < 0.05). The optimized MTX-NPs showed EE (%) of 86 ± 0.13 and DL (%) of 17 ± 0.32%.

### 3.4. FTIR Analysis of MTX-NPs and their Ingredients

**Figure 4:**
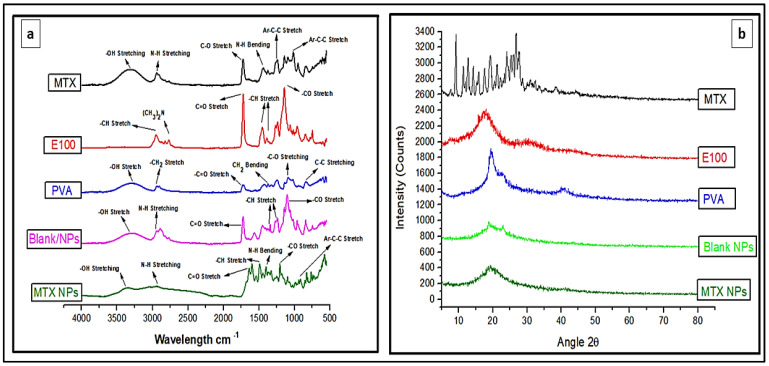
(**a**) FTIR spectra of MTX, E100, PVA, blank and MTX-NPs. (**b**) XRD spectra of MTX, E100, PVA, blank and MTX-NPs.

FTIR analysis identifies the chemical bonds in a molecule or material using infrared absorption spectrum. It is helpful to find any possible interaction among different constituents. FTIR spectra for MTX, E100, PVA, blank NPs and MTX-NPs was performed as depicted in Figure 4a, for any possible interaction. The pure MTX in FTIR spectra revealed a peak at 3306 cm^−1^, that was attributed to basic amine and hydroxyl (−NH and −OH) groups overlapping. The MTX band at 2940 cm^−1^ was recognized as symmetric/asymmetric stretching of −OH. The single strong peak at 1634 cm^−1^ that appeared in MTX was due to the carbonyl stretching vibrations by the overlapping of −NH bond. The characteristic stretching vibration of the aromatic ring skeleton appeared at 1441 cm^−1^. The additional two strong peaks seen at 1244 and 1012 cm^−1^ are the asymmetrical and symmetrical stretching of (COO−) groups. E100 showed a peak of ester group −CO at 1138 cm^−1^ and another strong ester (C=O) stretching band was observed at 1723 cm^−1^. The −CH groups were present at 1385, 1455 and 2947 cm^−1^. The dimethylamino group was present at 2761 cm^−1^. Blank NPs showed characteristic peak of dimethylamino group at 2944 cm^−1^, C=O stretching at 1726 cm^−1^ and −CH stretching at 1399 cm^−1^. FTIR spectra of MTX loaded NPs indicates the functional groups present in nanoparticles are almost same as in MTX and E100. In MTX-NPs, −OH is present at 3362 cm^−1^ and −CH stretching at 2933 cm^−1^, 1399 and 1202 cm^−1^ for COO− group while for E100 1645 cm^−1^ indicating C=O and 1491 cm^−1^ representing −CH group. All these FTIR spectra showed the successful preparation of NPs and incorporation of the drug into NPs without any chemical interaction of functional groups of the drug and the polymer.

### 3.5. XRD Analysis

XRD analysis was performed for the identification of the crystalline nature of a compound that helps in revealing the information about the chemical composition. MTX indicated sharp XRD peaks at 10, 20 and 28 θ, indicating that the MTX is crystalline in nature (Figure 4b). E100 showed peaks from 16 to 22 θ, while PVA showed a peak at 20 θ. The blank NPs with amorphous nature showed small peaks between 19 and 24 θ, but these peaks were not sharp. When MTX was incorporated into the NPs, a completely amorphous form was obtained, confirming that the drug was transformed from crystalline to amorphous form. The small crystallinity in MTX-NPs between 18 and 22 θ can be attributed to E100, which has a hydrophobic nature. Pure MTX exhibit multiple intense peaks. However, there were no characteristic peaks of MTX in the diffractogram of MTX-NPs. In addition, XRD patterns of blank NPs were similar to MTX-NPs, with no additional characteristic peaks suggesting that MTX was entrapped in E100 NPs as an amorphous or a disordered-crystalline state. Therefore, the characteristic patterns of crystalline MTX did not appear in MTX-NPs.

### 3.6. DSC Analysis

**Figure 5:**
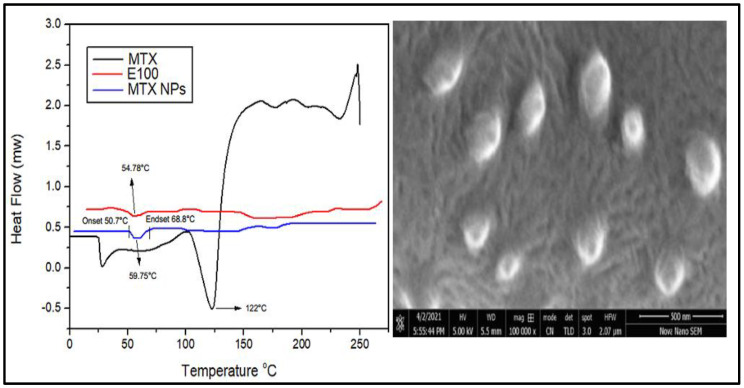
(**a**) Differential scanning calorimetry (DSC) thermograms of MTX, E100 and MTX-NPs. (**b**) Scanning electron microscope image of MTX-NPs.

DSC is a thermal analysis technique that involves measuring heat flow into and out of the sample as a function of temperature or time. DSC analysis provides information about the amorphous or crystalline nature of samples to confirm their physical properties. DSC thermograms of MTX, E100 and MTX-NPs are demonstrated in Figure 5a. DSC thermogram of MTX indicated a sharp endothermic peak at 122 °C, which corresponds to the melting peak of MTX [59]. E100 showed an endothermic peak at 54.78 °C matching to the transition temperature (Tg) of polymer. MTX-NPs presented a sharp endothermic peak at 59.75 °C, where the peaks of MTX were not observable, indicating that MTX was entangled into the NPs in either amorphous or disorderly crystalline form. MTX-NPs showed an onset glass transition temperature at 50.70 °C and endset at 68.8 °C with a mid-point at 59.75 °C as shown in Figure 5a.

### 3.7. Scanning Electron Microscopy (SEM) Analysis

SEM is used to obtain high resolution images of NPs to study the structure and any contamination or imperfection of the surface of NPs. Morphological studies were conducted using scanning electron microscopy. The SEM images disclosed the sphere-shaped of MTX-NPs (Figure 5b).

### 3.8. Characteristics of MTX-NPs Loaded Hydrogel

#### 3.8.1. Physical Appearance

A hydrogel should be clear, uniform and consistent in appearance. Chitosan hydrogel with 2% concentration was prepared and visually observed. The prepared chitosan hydrogel was clear, smooth, homogeneous and slightly yellowish in color. The hydrogel was uniform and there was no phase separation or lumps.

#### 3.8.2. Viscosity

**Figure 6:**
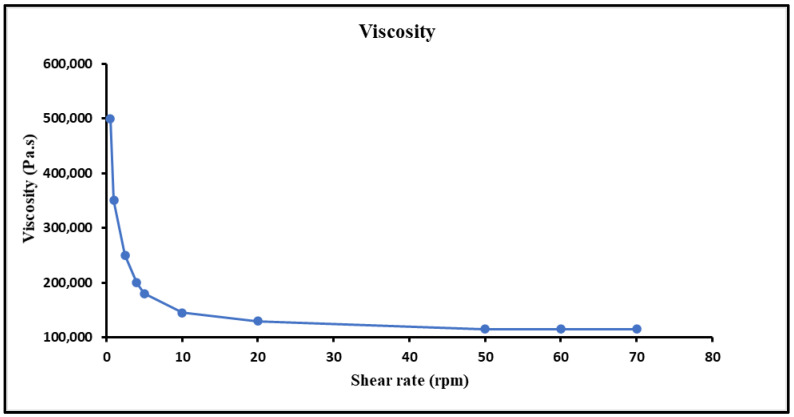
Viscosity of hydrogel at different shear rates and characterization, i.e., pH, extrudability, spreadability and drug content of MTX-NPs loaded hydrogel.

Appropriate viscosity of a hydrogel is of much importance with respect to the proper flow and spreading on skin. The viscosity of the prepared hydrogel was reduced when the shear rate was increased and showed a steady behaviour at higher shear rates, which indicated a shear thinning or pseudo plastic (non-Newtonian) behavior. This can be helpful since the hydrogel would become easier to spread and less painful upon application on the skin. Hydrogel can affect the adhesion to skin surface, so the rheological behaviour was performed, and curves were built between viscosities and shear rates. An inverse relationship of viscosity and shear rate was observed, i.e., viscosity was reduced by increasing the shear rate shown in Figure 6.

#### 3.8.3. Spreadability

The pseudo-plastic behaviour has been proven to boost the spreadability of hydrogel [60]. The spreadability profile test is the evaluation for uniform spreading of topical formulation [61]. It was noticed that by increasing the weight, the spreading area of the hydrogel was increased. The value of spreadability for the hydrogel was found to be 9.8 cm, which indicated that the hydrogel was easily spreadable by applying a slight amount of shear. This ensured that the hydrogel retained an excellent contact time at the site of application.

#### 3.8.4. Extrudability and pH of Hydrogel

Extrudability is the ability of material to be extruded out from a tube or nozzle by applying minimum energy. Prepared MTX-NPs loaded hydrogel showed an excellent extrudability of 18.28 g/cm^2^. The pH of MTX-NPs loaded hydrogel was found to be 6.4 ± 0.04, which is in an acceptable range for the topical formulations.

#### 3.8.5. Drug Content in Hydrogel

The drug content (%) in MTX-NPs loaded hydrogel was found to be 80.11 ± 0.21% indicating the uniform distribution the drug.

### 3.9. In Vitro Drug Release Studies

#### 3.9.1. In Vitro Drug Release from MTX-NPs and MTX-NPs Loaded Hydrogel

**Figure 7:**
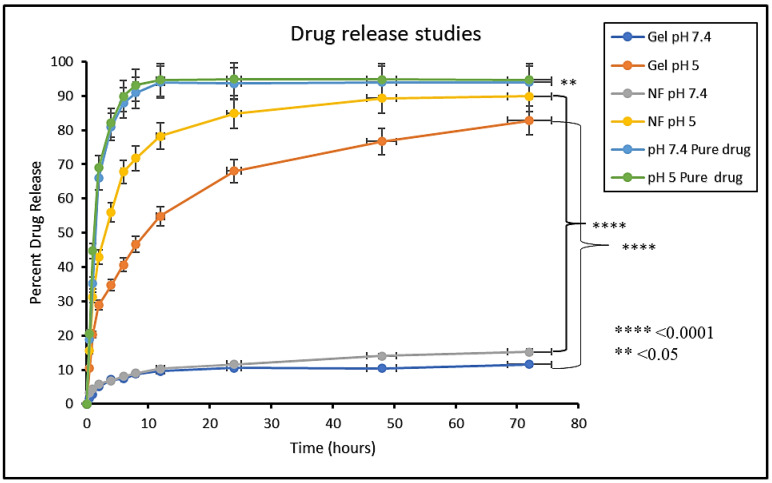
In vitro release study of MTX (Pure drug), MTX-NPs (NF) and MTX-NPs loaded hydrogel (gel) in phosphate buffers at various pH, i.e., 5 and 7.4, **** *p*-value ≤ 0.0001, ** *p*-value ≤ 0.05.

In vitro drug release study was performed to access the release of the drug from dosage forms under standardized in vitro conditions. In vitro release studies of MTX-NPs, MTX-NPs loaded hydrogel and free MTX as control was performed at pH 5 and 7.4 as shown in Figure 7. In case of free MTX, almost 90% of the drug was released within 4–6 h in both media. A burst release of the drug was shown by MTX-NPs at pH 5. Within 1 h, almost 31% of the drug was released and almost 71% of the drug was released within a time of 8 h. However, MTX-NPs loaded hydrogel showed a burst release of only 20% in 1 h, and a drug release of 46% in a sustained release pattern in 8 h and 73% in 48 h at pH 5, whereas the drug release from MTX-NPs and MTX-NPs loaded hydrogel at pH 7.4 was only 11 and 15% even in 72 h, respectively. The p value of 0.0001 shows a highly significant difference in drug release at different pH values (5 and 7.4) in both MTX-NPs and MTX-NPs loaded hydrogel, while less significant (*p* < 0.05) correlation has been observed in pure drug solution at pH (5 and 7.4).

#### 3.9.2. Kinetics Models for Drug Release from NPs and Hydrogel

Various kinetic models, i.e., zero order, first order, Higuchi, Korsemeyer-Peppas and Hixson-Crowell were implied using DD solver software to calculate drug release, its mechanism and diffusion characteristics from MTX-NPs and MTX-NPs loaded hydrogel. The model having the highest R^2^ value was chosen as Best-fit model. Drug release from MTX-NPs at pH (5 and 7.4) having R^2^ values 0.9944 and 0.9952, respectively showed the greatest linearity for Higuchi model, which describes a sustained release of MTX from polymer (E100) based on diffusion control. The n value observed was 0.301 and 0.223 for MTX released from NPs at pH 5 and 7.4, respectively. The value of n less than 0.45 suggests a Fickian diffusion-based release of MTX from polymer. Likewise, MTX release from MTX-NPs loaded hydrogel at selected pH (5 and 7.4) having R^2^ values 0.999 and 0.9984 showed the maximum linearity for Higuchi model as compared to other models presented in Table S1, (Supplementary Materials).

### 3.10. Cell Viability

**Figure 8:**
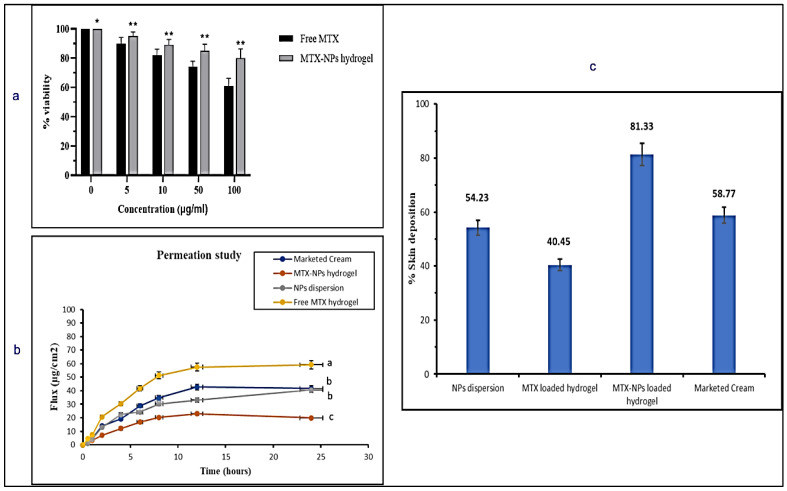
(**a**) In vitro cell viability assay in blood lymphocytes treated with free MTX and MTX-NPs loaded hydrogel. Results are indicated as mean ± SD, n = 3. * *p* < 0.05, ** *p* < 0.01, (**b**) Percent cumulative drug permeation of free MTX hydrogel, NPs dispersion, MTX-NPs hydrogel and marketed cream. Data has been represented as mean ± SD where different superscripts (**a**–**c**) represent significant difference (*p* < 0.05) between various formulations, (**c**) Percent skin deposition of free MTX hydrogel, NPs dispersion, MTX-NPs hydrogel and marketed cream.

Cell viability assay is performed to measure proportion of healthy and live cells in experimental conditions and treatment conditions. The percentage of viable cells acquired from blood lymphocytes after treating with free drug (MTX) and MTX-NPs loaded hydrogel is presented in Figure 8a. With an increase in the quantity of MTX from 0 to 100 µg/mL, the percentage of viable cells was considerably decreased from 100 to 61%. On the contrary, when the cell viability was evaluated against MTX-NPs loaded hydrogel, it was found to be significantly higher, i.e., 80% at 100 µg/mL concentration in comparison to free MTX. If cell viability is greater than 70%, it is considered to be safe. The developed MTX-NPs loaded hydrogel displayed 80% cell viability, which confirmed its safety and biocompatibility.

### 3.11. Ex Vivo Skin Permeation Studies

An ex vivo skin permeation study was performed to mimic the conditions of skin to check efficacy of the developed formulation. The permeation profile of the drug was investigated for marketed cream, MTX-NPs loaded hydrogel, NPs dispersion and free MTX hydrogel for a period of 24 h on the excised skin of mice using Franz diffusion cell. The MTX-NPs loaded hydrogel showed only 19.95 ± 51.04 µg/cm^2^ permeation through the skin, whereas NPs dispersion demonstrated 40.65 ± 51.13 µg/cm^2^ of drug permeation after 24 h. The marketed cream exhibited 41.63 ± 0.12 µg/cm^2^ permeation. Free MTX hydrogel showed the highest permeation of 59.23 ± 0.34 µg/cm^2^ depicted in Figure 8b. MTX-NPs loaded hydrogel showed lower permeation in comparison to other formulations.

### 3.12. Skin Deposition Studies

It is very important to check how much drug is retained or deposited in the skin for evaluating the efficacy of a topical dosage form. It is very critical to treat the psoriatic skin because of hyper-proliferation in the epidermal keratinocytes, which not only limits the use of transdermal formulation but suggests formulations for local application [62]. So, it is very important for the ideal formulation to deposit the drug in the epidermal layers of the skin for a longer span of time to release the drug after application with minimal permeation. Skin deposition for marketed cream, MTX-NPs loaded hydrogel, NPs dispersion and free MTX hydrogel was performed where MTX-NPs loaded hydrogel showed the highest deposition of drug (81%) in the epidermal skin layer in comparison to other formulations as depicted in Figure 8c. Thus, MTX-NPs loaded hydrogel formulation was observed to be the ideal for topical management of psoriasis.

### 3.13. Skin Irritation Studies

**Figure 9:**
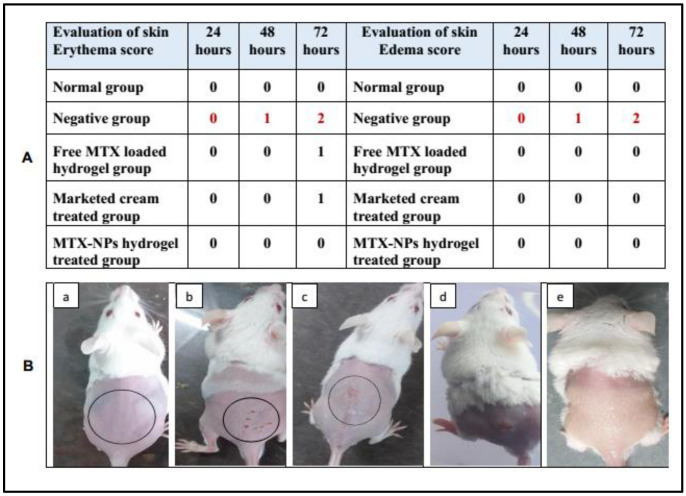
(**A**) Skin irritation scores for erythema and edema at 24, 48 and 72 h, (**B**) Visual representation of skin irritation for different treatments, i.e., (**a**) Normal group (**b**) 0.8% formalin treated as a negative control, (**c**) Treated with free MTX-loaded hydrogel, (**d**) Treated with marketed cream (**e**) Treated with MTX-NPs loaded hydrogel.

Skin irritation is an in vitro test performed to check any possible irritation caused by a developed formulation when applied to the skin. MTX-NPs loaded hydrogel was observed to be non-irritant to the skin. While in free MTX hydrogel and marketed cream some signs of erythema were noticed. Similarly, only the negative group showed signs of edema, while no edema was observed in other groups. The results of the initial skin irritation findings are presented in Figure 9A, and the photographs are illustrated in Figure 9B, (a–e). Draize patch test is a steadfast procedure as the results found from this irritation study can be related to those in human beings [49].

### 3.14. In Vivo Antipsoriatic Studies and Scoring Severity of Skin Inflammation

**Figure 10:**
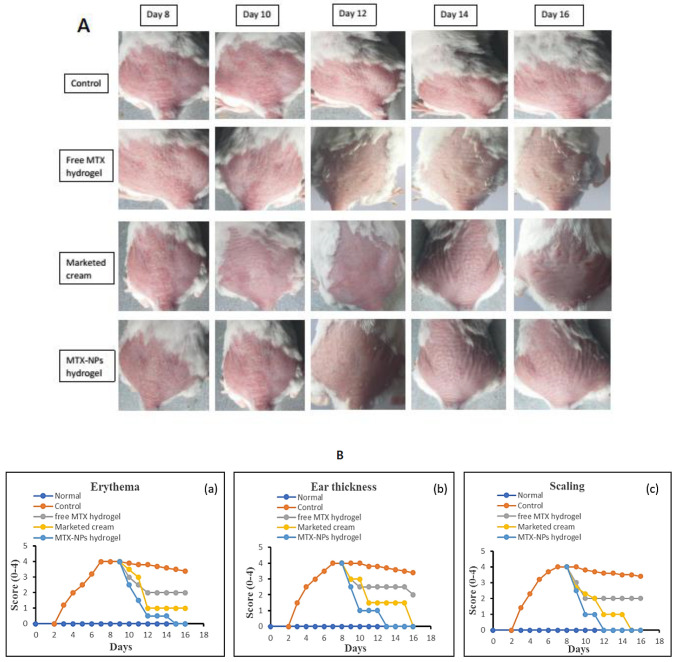
(**A**) Antipsoriatic activity of various formulations (free MTX hydrogel, marketed cream & MTX-NPs hydrogel) in IMQ-induced mice in comparison with control (with no treatment) (n = 5). (**B**) (**a**–**c**) showing assessment of psoriasis severity index score of various formulations treatment, i.e., (free MTX hydrogel, marketed cream & MTX-NPs hydrogel) in IMQ-induced mice in comparison with control (with no treatment) (n = 5).

The in vivo effectiveness of the MTX-NPs loaded hydrogel formulation was evaluated in BALB/c mice through IMQ-induced psoriasis. The therapeutic efficiency of MTX-NPs loaded hydrogel was evaluated in comparison with free MTX hydrogel and marketed cream. Figure 10, showing the psoriasis treatment using free MTX hydrogel, marketed cream and MTX-NPs loaded hydrogel for 8 consecutive days. The control group (no treatment) and other three treated groups showed psoriasis-like signs, i.e., redness, inflammation and thickness of skin at day 0 of treatment, as shown in Figure 10A. From day 8 to onwards a quicker improvement was noticed for psoriatic signs in group of mice treated with MTX-NPs loaded hydrogel as compared to the free MTX hydrogel and marketed cream showed in Figure 10A. The psoriatic signs started to disappear from day 10 from the group of mice treated with MTX-NPs loaded hydrogel as compared to free MTX hydrogel and marketed cream. The free MTX hydrogel showed less effectiveness as compared to MTX-NPs loaded hydrogel as well as marketed cream because it was not able to completely heal the psoriatic-like signs after 8 days of treatment therapy. This fact can be related to the poor penetration of MTX from hydrogel following topical application.

Figure 10B, shows the severity index scores for psoriasis. Imiquimod cream was gently applied on shaved dorsal skin and on the right ear of mice for 7 successive days on all groups, while group 1 was kept as normal. After applying IMQ cream, from day 2, the initial signs of erythema, scaling and ear thickness started to appear in all groups of mice, except group 1. From day 3 onwards, the signs of scaling and erythema started to progress, with an increase in the severity. The thickness of the right ear was measurable from day 3 onwards, with advancement in the severity as compared to the left ear, which was used as control. It was observed that PASI score increased in all groups from 2 to 5 with application of Imiquimod (IMQ) cream. After 7 days of IMQ application, the treatment started from day 8 to 16 with different formulations in group 3 to 5. Group 3 was given treatment with free MTX hydrogel, group 4 with marketed cream and group 5 with MTX-NPs loaded hydrogel. The severity and PASI score of erythema, scaling and ear thickness resulted in a decrease in all groups, but a marked decrease was noticed in group 5 treated with MTX-NPs loaded hydrogel, as compared to marketed cream and free MTX hydrogel, as depicted in Figure 10B.

### 3.15. Histopathological Studies

**Figure 11:**
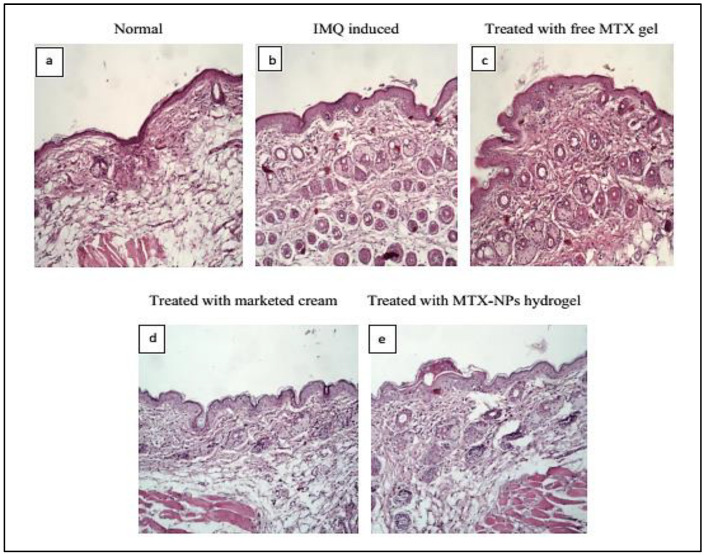
Histopathology of BALB/c mice skin after 10 days of topical treatment (**a**) Normal (**b**) IMQ induced (**c**) Treated with free MTX hydrogel (**d**) Treated with marketed cream (**e**) treated with MTX-NPs loaded hydrogel.

Histopathology is the study to diagnose disease at tissue level by examining the cell or tissue under microscope. The H&E staining for healthy mice skin displayed normal epithelium with intact epidermis. In the top layer of the epidermis, there was a keratin layer in a well-formed structure without any signs of inflamed cells. Psoriatic skin showed erythema, elongated rete ridges, acanthosis and hyperplasia with leukocytes infiltration in the epidermis and dermis region. The free MTX hydrogel treated group demonstrated a decrease in the thickness of both the dermis and epidermis, while leukocytic infiltrations were present. The group treated with marketed cream showed a noticeable reduction in psoriatic lesions with better hyperkeratosis and parakeratosis results. The group treated with MTX-NPs loaded hydrogel displayed a healthy keratin layer with decreased parakeratosis and hyperkeratosis level as depicted in Figure 11. These observations revealed that MTX-NPs loaded hydrogel was highly effective in mitigating psoriatic symptoms in IMQ induced mice models.

### 3.16. Spleen to Body Weight Percent (Spleen/Body Weight %)

**Figure 12:**
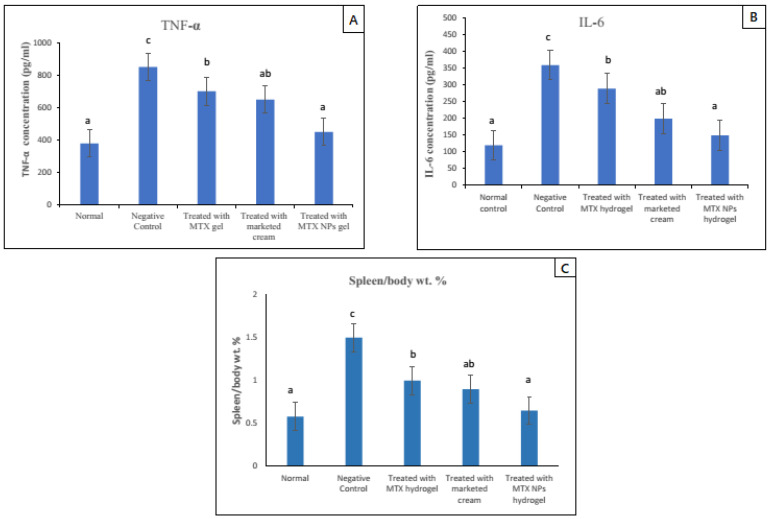
TNF-*α* and IL–6 levels in blood serum of mice from various treated groups in comparison to normal group, data has been represented as mean ± SD where different superscripts (**A**–**C**) represent significant difference (*p* < 0.05) between various formulations i.e., a representing normal and treated with MTX-NPs loaded hydrogel, ab, group treated with marketed cream, b, treated with free MTX hydrogel and c, no treatment. Spleen to body weight was calculated after mice were sacrificed. Data has been represented as mean ± SD where different superscripts (**A**–**C**) represent significant difference (*p* < 0.05) between various formulations.

Due to the activation of the immune system in diseases such as psoriasis, the spleen cells increase in number, ultimately increasing the whole spleen weight. The spleen/body weight percent was calculated for psoriasis-induced (negative control group) and normal group, and it was nearly three times higher, which indicated a rise in the number of spleen cells. Subsequently, this finding indicated activation of immune response in the psoriasis mice model. In comparison to the negative group, all other groups that were given treatment with free MTX hydrogel, marketed cream and MTX-NPs loaded hydrogel were evaluated for spleen/body weight percent values. Among all these groups, the lowest value for spleen/body weight percent was noticed in the MTX-NPs loaded hydrogel treated group, as its value was close to the normal group. The free MTX hydrogel treated group demonstrated a similar outcome to the marketed cream treated group (*p* < 0.05). Furthermore, there was no noticeable variation between the normal and MTX-NPs loaded hydrogel treated groups on spleen/body weight percent (*p* > 0.05) as depicted in Figure 12C.

### 3.17. TNF-α and IL–6 Assessment in Skin

The increase in pro-inflammatory cytokines is a sign of hyperproliferation in certain disease conditions. Potent pro-inflammatory cytokines such as TNF-*α* and IL–6 are capable of inducing proliferation activities and play a major role in the pathogenesis of psoriasis [63]. In psoriasis, IL–6 production is increased, which synergizes TNF-*α* and IL–1 to advance the hyperproliferation of epidermis by its activity on epidermal growth factor receptor (48). The ELISA technique was used to determine the levels of TNF-α and IL–6 in mice skin of various groups. The elevated level of both TNF-α and IL–6 was noticed in the case–control group (negative control) as compared with the normal group. While in groups in which psoriasis was induced and treated with different formulations showed reduced levels of both TNF-α and IL–6. The group treated with MTX-NPs loaded hydrogel showed approximately 84% (400 pg/mL) decrease in TNF-α and 80% (210 pg/mL) decrease in IL–6. There was approximately 58% (200 pg/mL) decrease in TNF-α and 60% (160 pg/mL) decrease in IL–6 levels in group treated with marketed cream as depicted in Figure 12 A,B. The developed formulation of MTX-NPs loaded hydrogel showed better results in the reduction of both of TNF-*α* and IL–6, as compared to free MTX hydrogel and marketed cream.

### 3.18. Stability Studies

A stability study was performed to check the physical appearance, clarity, consistency, uniformity, particle size and pH of the pharmaceutical product. This study ensures the maintenance of safety, efficacy and quality of prepared formulation during the shelf life [64]. These studies are required to be conducted according to the guidelines of ICH [65]. These guidelines were followed to conduct the accelerated stability studies to check the effect of relative humidity (%RH) and temperature, i.e., 4 ± 2 °C, 25 ± 52 °C and 40 ± 2 °C/75% RH on final nanoformulation and hydrogel over a period of 6 months. Particle size and encapsulation efficiency of MTX-NPs while pH, drug content and spreadability of hydrogel were evaluated. Particle size of MTX-NPs was a bit increased due to aggregation while EE was decreased from 86 to 81%. Similarly in case of hydrogel, no noticeable change in pH was observed, a slight decrease in drug content from 81.7 ± 0.2 to 78.2 ± 0.3%, while spreadability decreased from 11.4 ± 0.2 to 8.2 ± 0.1. Both the nanoformulation and hydrogel retained their properties, and no phase separation was observed. The results obtained from these studies revealed the stability of MTX-NPs and MTX-NPs loaded hydrogel (Table 2).

The main focus of this study was to develop a nanocarrier system that could not only deliver the drug to the target site, but also keep the drug in the topical region. Topical drug delivery techniques are preferred for the management of localized diseases. This is an appealing delivery strategy against psoriasis as the initiation and advancement of this disease mainly takes place in the skin. In this work, MTX-NPs were formulated and assessed for the topical management of psoriasis. For appropriate and homogeneous topical application of MTX-NPs, Chitosan hydrogel was used as a matrix for the NPs, which not only ensured the consistency of formulation, but also extended the contact time of drug on the skin. MTX-NPs were developed to target psoriasis. Systemically, MTX, which is effective against various dermatological conditions, have adverse events when used in patients with localized disease. In certain cutaneous inflammatory disorders such as psoriasis, the hyperplasia of the skin is managed by the topical application of drugs such as MTX. E100, a pH sensitive polymer that degrades at skin pH, which was used to prepare NPs for topical delivery of MTX. The prepared MTX-NPs were then loaded in the chitosan hydrogel which is a natural, nontoxic, biodegradable and bio-compatible polymer [66]. Chitosan has a natural pH-responsive properties due to the protonation-deprotonation balance of amino groups [67]. E100 does not have intrinsic physical or chemical instability and due to the presence of a tertiary amino group, the neutral pH of skin (4–6) affects the integrity of the copolymer [68]. It is very important for topical medication that the total amount of drug should be released on the skin.

The measurements of average particle size and PDI of optimized MTX-NPs by dynamic light scattering indicated narrow size distribution. In common, PDI is the measurement of particles distribution from 0.0 and 0.5 representing narrow and very wide distributions, respectively [69]. The uniformity and narrow size distribution of NPs was confirmed by SEM analysis. PDI values of 0.2 or to a lesser extent are normally satisfactory for polymer based NPs [70]. The value of zeta potential is the measure of charge at the surface of nanoparticles which indicates physical stability of particles within the colloidal system. The measurement of positive charge on blank and drug loaded NPs revealed the inherent stability properties of polymer as it has cationic nature.

Characteristic peaks of E100 were noticed in FTIR spectra of blank NPs, with no significant extra peaks. Similarly, the spectra of MTX-loaded NPs had similar absorption peaks to that of MTX and E100, with no extra peaks. Therefore, it was concluded that there were no signs of chemical interface between MTX and the polymer. In XRD analysis there were no extra peaks in MTX-loaded NPs, and this was similar to the blank NPs. The amorphous form of MTX-loaded NPs suggested that MTX was completely entrapped in E100 NPs, hence there was no crystalline pattern [71]. DSC thermograms of MTX-loaded NPs displayed a characteristic endothermic peak of E100 at 54.78 °C. Therefore, MTX had no detectable melting peak, which showed that MTX was perfectly encapsulated E100 NPs in an amorphous form. The crystalline form of MTX was not observed in NPs which was confirmed by the absence of characteristic endothermic peak which was observed separately at 122 °C. SEM images confirmed the spherical shape of nanoparticles and affirmed the particle size in accordance to DLS results.

The in vitro drug release study is vital tool to evaluate the dissolution, solubility and drug release from NPs in comparison to pure drug. The release profile of pure MTX and prepared NPs and NPs loaded hydrogel were evaluated in phosphate buffer media. The release kinetics clearly revealed the diffusion control is the basic mechanism of drug release. Furthermore, R^2^ value (0.99) was with respect to the Higuchi model and the n value indicated the mode of diffusion was Fickian transport [72]. Skin permeation and deposition studies revealed that the drug was retained in the layers of skin which is ideal for the topical formulations.

The prepared hydrogel in which NPs were loaded revealed a decline in viscosity with raising the shear rate that is specific property of shear thinning behaviour which makes hydrogel suitable for topical applications [1]. The MTX-NPs loaded hydrogel after application on the skin, released the drug from pH sensitive polymer after equilibrating with the skin’s natural pH by breaking down the polymer to release drug ingredients. This ensured the release of entire concentration of drug from nanoparticles. This nanocarrier system was evaluated for skin deposition and it was found that NPs loaded in hydrogel remained intact in the epidermis layers of skin after 24 h [73].

In vivo studies revealed that psoriasis induced in mice was successfully treated by the nanocarrier system. The plaque formation was confirmed by H & E staining which provided evidence of erythema, desquamation and thickness of skin. The MTX-loaded NPs hydrogel significantly relieved all the psoriatic symptoms as compare to the positive control, free MTX hydrogel and marketed cream. Moreover, spleen/body weight percent and PASI score suggested good therapeutic efficiency. In IMQ-induced psoriatic mice model, IL–6 and TNF-*α* were reduced noticeably in MTX-loaded hydrogel treated group which further confirmed the efficacy of developed formulation.

## 4. Conclusions

A nanocarrier system which relies on the skin’s natural pH was developed. Methotrexate was encapsulated into pH sensitive polymeric NPs of E100, and the prepared NPs were loaded into the hydrogel. MTX-NPs were optimized for the enhancement of solubility and better skin deposition to gain the better therapeutic benefits of MTX to manage psoriasis. Chitosan-based hydrogel was formulated to load the MTX-NPs for more effective delivery of MTX. MTX-NPs loaded hydrogel was stable during 90 days of physical stability test, with no significant changes in appearance, pH, drug content and viscosity. The Higuchi kinetic release model was followed with a diffusion control mechanism, demonstrating sustained drug release from the nanocarrier system. In vivo results showed that psoriasis was induced in the mice model and successfully treated, which was also confirmed by H&E staining. It can be suggested that the developed MTX-NPs loaded hydrogel system gives the impression that it is a better approach to enhance the topical effectiveness of MTX for psoriasis management.

## Figures and Tables

**Table 1:** Optimization of MTX-loaded NPs.

S No.	E100 (mg)	PVA (mg)	MTX (mg)	P Size (nm)	PDI	Zeta Potential (mV)	EE (%)	DL (%)
1	112.5	97.5	3	137	0.15	43.7	66	14
2	112.5	25	4	165	0.10	42.0	69	15
3	112.5	25	6	255	0.17	26.4	86	17
4	112.5	170	6	150	0.12	36.5	77	16
5	112.5	97.5	4	137	0.15	43.7	75	14
6	200	97.5	3	256	1.00	36.4	62	10
7	200	25	4	323	0.21	19.3	66	12
8	25	170	4	75	0.16	27.0	25	6.54
9	25	97.5	3	87	0.27	34.6	23	8.33
10	25	25	4	97	0.15	34.2	60	11.53
11	112.5	97.5	4	137	0.15	43.7	75	15
12	25	97.5	6	89	0.13	21.3	62	12.5
13	200	170	4	112	0.14	33.2	62	13.33
14	200	97.5	6	165	0.10	42.0	76	14.28
15	112.5	170	3	149	0.17	40.7	75	7.69

**Table 2:** Stability studies of NPs and hydrogel discussion.

	MTX-NPs									MTX-NPs Loaded Hydrogel								
Time in Months	Particle Size (nm)			Encapsulation Efficiency (%)			Phase Separation			pH			Drug Content (%)			Spreadability (cm/sec)		
Temp ±2 °C	4	25	40	4	25	40	4	25	40/75% RH	4	25	40/75% RH	4	25	40/75% RH	4	25	40/75% RH
0	256 ± 2.17	256 ± 2.17	256 ± 2.12	86 ± 0.03	86 ± 0.05	86 ± 0.13	NO	NO	NO	6.4 ± 0.2	6.4 ± 0.4	6.4 ± 0.6	81.7 ± 0.2	81.4 ± 0.6	81.2 ± 0.4	11.4 ± 0.2	11.2 ± 0.5	11.5 ± 0.1
1	256 ± 2.07	256 ± 2.17	256 ± 2.13	85 ± 0.13	85 ± 0.15	85 ± 0.11	NO	NO	NO	6.4 ± 0.12	6.4 ± 0.14	6.4 ± 0.16	81.7 ± 0.02	81.4 ± 0.06	81.2 ± 0.04	10.3 ± 0.3	10.4 ± 0.4	10.6 ± 0.6
3	258 ± 1.23	256 ± 1.27	256 ± 1.22	84 ± 0.23	84 ± 0.15	84 ± 0.17	NO	NO	NO	6.3 ± 0.6	6.2 ± 0.3	6.4 ± 0.6	80.3 ± 0.1	80.5 ± 0.4	80.3 ± 0.6	9.8 ± 0.1	9.8 ± 0.1	9.8 ± 0.1
6	257 ± 1.12	257 ± 1.16	257 ± 1.18	81 ± 0.21	81 ± 0.025	81 ± 0.19	NO	NO	NO	6.6 ± 0.1	6.8 ± 0.5	6.5 ± 0.3	78.2 ± 0.3	78.4 ± 0.5	78.5 ± 0.2	8.2 ± 0.1	8.3 ± 0.4	8.5 ± 0.6

## Data Availability

The data are included in the manuscript.

## Supplementary Materials

The following are available online at https://www.mdpi.com/article/10.3390/nano11123433/s1, Table S1: Kinetics models for drug release from NPs and hydrogel.

## References

[1] Walunj M., Doppalapudi S., Bulbake U., Khan W. (2020). Preparation, characterization, and in vivo evaluation of cyclosporine cationic liposomes for the treatment of psoriasis. J. Liposome Res..

[2] Gudjonsson J., Johnston A., Sigmundsdottir H., Valdimarsson H. (2004). Immunopathogenic mechanisms in psoriasis. Clin. Exp. Immunol..

[3] Lacarrubba F., Pellacani G., Gurgone S., Verzì A.E., Micali G. (2015). Advances in non-invasive techniques as aids to the diagnosis and monitoring of therapeutic response in plaque psoriasis: A review. Int. J. Dermatol..

[4] Singhvi G., Hejmady S., Rapalli V.K., Dubey S.K., Dubey S. (2020). Nanocarriers for Topical Delivery in Psoriasis. Delivery of Drugs.

[5] Vincent N., Ramya D.D., Vedha H.B. (2014). Progress in psoriasis therapy via novel drug delivery systems. Dermatol. Rep..

[6] Mehnert W., Mäder K. (2012). Solid lipid nanoparticles: Production, characterization and applications. Adv. Drug Deliv. Rev..

[7] Gupta M., Agrawal U., Vyas S.P. (2012). Nanocarrier-based topical drug delivery for the treatment of skin diseases. Expert Opin. Drug Deliv..

[8] Patra J.K., Das G., Fraceto L.F., Campos E.V.R., del Pilar Rodriguez-Torres M., Acosta-Torres L.S., Diaz-Torres L.A., Grillo R., Swamy M.K., Sharma S. (2018). Nano based drug delivery systems: Recent developments and future prospects. J. Nanobiotechnology.

[9] Ganguly S., Das P., Itzhaki E., Hadad E., Gedanken A., Margel S. (2020). Microwave-synthesized polysaccharide-derived carbon dots as therapeutic cargoes and toughening agents for elastomeric gels. ACS Appl. Mater. Interfaces.

[10] Kurian A., Barankin B. (2011). Current effective topical therapies in the management of psoriasis. Ski. Ther. Lett..

[11] Sun L., Liu Z., Wang L., Cun D., Tong H.H., Yan R., Chen X., Wang R., Zheng Y. (2017). Enhanced topical penetration, system exposure and anti-psoriasis activity of two particle-sized, curcumin-loaded PLGA nanoparticles in hydrogel. J. Control. Release.

[12] Kaur N., Sharma K., Bedi N. (2018). Topical nanostructured lipid carrier based hydrogel of mometasone furoate for the treatment of psoriasis. Pharm. Nanotechnol..

[13] Asad M.I., Ahmed N., Rehman A.u., Khan G.M. (2019). Polylactide: The polymer revolutionizing the biomedical field. Mater. Biomed. Eng..

[14] Nikam V.K., Kotade K., Gaware V., Dolas R., Dhamak K., Somwanshi S., Khadse A., Kashid V. (2011). Eudragit a versatile polymer: A review. Pharmacologyonline.

[15] Vijaya R., Maheshwari U., Bharathi J. (2015). Development and in vitro evaluation of Eudragit E100 and PVP based matrix films for the transdermal delivery of Repaglinide. Pharma Innov. J..

[16] Jang J.-H., Jeong S.-H., Lee Y.-B. (2019). Preparation and in vitro/in vivo characterization of polymeric nanoparticles containing methotrexate to improve lymphatic delivery. Int. J. Mol. Sci..

[17] Aickara D., Bashyam A.M., Pichardo R.O., Feldman S.R. (2020). Topical methotrexate in dermatology: A review of the literature. J. Dermatol. Treat..

[18] Van Roon E., Van de Laar M. (2010). Methotrexate bioavailability. Clin. Exp. Rheumatol.-Incl. Suppl..

[19] Qindeel M., Khan D., Ahmed N., Khan S., Rehman A.u. (2020). Surfactant-free, self-assembled nanomicelles-based transdermal hydrogel for safe and targeted delivery of methotrexate against rheumatoid arthritis. ACS Nano.

[20] Abolmaali S.S., Tamaddon A.M., Dinarvand R. (2013). A review of therapeutic challenges and achievements of methotrexate delivery systems for treatment of cancer and rheumatoid arthritis. Cancer Chemother. Pharmacol..

[21] Guzmán M., Manzo R., Olivera M. (2012). Eudragit E100 as a drug carrier: The remarkable affinity of phosphate ester for dimethylamine. Mol. Pharm..

[22] Doerdelmann G., Kozlova D., Epple M. (2014). A pH-sensitive poly(methyl methacrylate) copolymer for efficient drug and gene delivery across the cell membrane. J. Mater. Chem. B.

[23] Sabir F., Asad M.I., Qindeel M., Afzal I., Dar M.J., Shah K.U., Zeb A., Khan G.M., Ahmed N., Din F.-U. (2019). Polymeric nanogels as versatile nanoplatforms for biomedical applications. J. Nanomater..

[24] Mir M., Ahmed N., Permana A.D., Rodgers A.M., Donnelly R.F., Rehman A.u. (2019). Enhancement in site-specific delivery of carvacrol against methicillin resistant Staphylococcus aureus induced skin infections using enzyme responsive nanoparticles: A proof of concept study. Pharmaceutics.

[25] Khalid A., Ahmed N., Qindeel M., Asad M.I., Khan G.M., Rehman A.u. (2021). Development of novel biopolymer-based nanoparticles loaded cream for potential treatment of topical fungal infections. Drug Dev. Ind. Pharm..

[26] Wu F., Meng G., He J., Wu Y., Wu F., Gu Z. (2014). Antibiotic-loaded chitosan hydrogel with superior dual functions: Antibacterial efficacy and osteoblastic cell responses. ACS Appl. Mater. Interfaces.

[27] Sami A.J., Khalid M., Jamil T., Aftab S., Mangat S.A., Shakoori A., Iqbal S. (2018). Formulation of novel chitosan guargum based hydrogels for sustained drug release of paracetamol. Int. J. Biol. Macromol..

[28] Bhattarai N., Gunn J., Zhang M. (2010). Chitosan-based hydrogels for controlled, localized drug delivery. Adv. Drug Deliv. Rev..

[29] Sohrabi S., Haeri A., Mahboubi A., Mortazavi A., Dadashzadeh S. (2016). Chitosan gel-embedded moxifloxacin niosomes: An efficient antimicrobial hybrid system for burn infection. Int. J. Biol. Macromol..

[30] Ravi P.R., Vats R., Dalal V., Gadekar N. (2015). Design, optimization and evaluation of poly-ɛ-caprolactone (PCL) based polymeric nanoparticles for oral delivery of lopinavir. Drug Dev. Ind. Pharm..

[31] Mir M., Permana A.D., Ahmed N., Khan G.M., Rehman A.u., Donnelly R.F. (2020). Enhancement in site-specific delivery of carvacrol for potential treatment of infected wounds using infection responsive nanoparticles loaded into dissolving microneedles: A proof of concept study. Eur. J. Pharm. Biopharm..

[32] Das P., Maity P.P., Ganguly S., Ghosh S., Baral J., Bose M., Choudhary S., Gangopadhyay S., Dhara S., Das A.K. (2019). Biocompatible carbon dots derived from κ-carrageenan and phenyl boronic acid for dual modality sensing platform of sugar and its anti-diabetic drug release behavior. Int. J. Biol. Macromol..

[33] Abdel-Salam F.S., Elkheshen S.A., Mahmoud A.A., Ammar H.O. (2016). Diflucortolone valerate loaded solid lipid nanoparticles as a semisolid topical delivery system. Bull. Fac. Pharm. Cairo Univ..

[34] Sachan A.K., Gupta A., Arora M. (2016). Formulation & characterization of nanostructured lipid carrier (NLC) based gel for topical delivery of etoricoxib. J. Drug Deliv. Ther..

[35] Cascone M.G., Lazzeri L., Carmignani C., Zhu Z. (2002). Gelatin nanoparticles produced by a simple W/O emulsion as delivery system for methotrexate. J. Mater. Sci. Mater. Med..

[36] Balzus B., Sahle F.F., Hönzke S., Gerecke C., Schumacher F., Hedtrich S., Kleuser B., Bodmeier R. (2017). Formulation and ex vivo evaluation of polymeric nanoparticles for controlled delivery of corticosteroids to the skin and the corneal epithelium. Eur. J. Pharm. Biopharm..

[37] Avasatthi V., Pawar H., Dora C.P., Bansod P., Gill M.S., Suresh S. (2016). A novel nanogel formulation of methotrexate for topical treatment of psoriasis: Optimization, in vitro and in vivo evaluation. Pharm. Dev. Technol..

[38] Zhang L., Xue J., Zhou X., Fei X., Wang Y., Zhou Y., Zhong L., Han X. (2014). Adsorption of molybdate on molybdate-imprinted chitosan/triethanolamine gel beads. Carbohydr. Polym..

[39] Fiume M.M., Heldreth B., Bergfeld W.F., Belsito D.V., Hill R.A., Klaassen C.D., Liebler D., Marks J.G., Shank R.C., Slaga T.J. (2013). Safety assessment of triethanolamine and triethanolamine-containing ingredients as used in cosmetics. Int. J. Toxicol..

[40] Karade P. (2012). Formulation and evaluation of celecoxib gel. J. Drug Deliv. Ther..

[41] Bachhav Y.G., Patravale V.B. (2009). Microemulsion based vaginal gel of fluconazole: Formulation, in vitro and in vivo evaluation. Int. J. Pharm..

[42] Qindeel M., Ahmed N., Sabir F., Khan S., Rehman A.u. (2019). Development of novel pH-sensitive nanoparticles loaded hydrogel for transdermal drug delivery. Drug Dev. Ind. Pharm..

[43] Shin S.-B., Cho H.-Y., Kim D.-D., Choi H.-G., Lee Y.-B. (2010). Preparation and evaluation of tacrolimus-loaded nanoparticles for lymphatic delivery. Eur. J. Pharm. Biopharm..

[44] Majid M., Nasir B., Zahra S.S., Khan M.R., Mirza B., Haq I.-U. (2018). *Ipomoea batatas* L. Lam. ameliorates acute and chronic inflammations by suppressing inflammatory mediators, a comprehensive exploration using in vitro and in vivo models. BMC Complementary Altern. Med..

[45] Phull A.-R., Eo S.-H., Abbas Q., Ahmed M., Kim S.J. (2016). Applications of chondrocyte-based cartilage engineering: An overview. BioMed Res. Int..

[46] Khan D., Qindeel M., Ahmed N., Khan A.U., Khan S., Rehman A.u. (2020). Development of novel pH-sensitive nanoparticle-based transdermal patch for management of rheumatoid arthritis. Nanomedicine.

[47] Algahtani M.S., Ahmad M.Z., Ahmad J. (2020). Nanoemulsion loaded polymeric hydrogel for topical delivery of curcumin in psoriasis. J. Drug Deliv. Sci. Technol..

[48] Algahtani M.S., Ahmad M.Z., Nourein I.H., Ahmad J. (2020). Co-delivery of imiquimod and curcumin by nanoemugel for improved topical delivery and reduced psoriasis-like skin lesions. Biomolecules.

[49] Patel M.R., Patel R.B., Parikh J.R., Patel B.G. (2016). Novel isotretinoin microemulsion-based gel for targeted topical therapy of acne: Formulation consideration, skin retention and skin irritation studies. Appl. Nanosci..

[50] Jain A., Doppalapudi S., Domb A.J., Khan W. (2016). Tacrolimus and curcumin co-loaded liposphere gel: Synergistic combination towards management of psoriasis. J. Control. Release.

[51] Chandra A., Aggarwal G., Manchanda S., Narula A. (2019). Development of topical gel of methotrexate incorporated ethosomes and salicylic acid for the treatment of psoriasis. Pharm. Nanotechnol..

[52] Pandey S.S., Shah K.M., Maulvi F.A., Desai D.T., Gupta A.R., Joshi S.V., Shah D.O. (2021). Topical delivery of cyclosporine loaded tailored niosomal nanocarriers for improved skin penetration and deposition in psoriasis: Optimization, ex vivo and animal studies. J. Drug Deliv. Sci. Technol..

[53] De Porto A., Lammers A., Bennink R., Ten Berge I., Speelman P., Hoekstra J. (2010). Assessment of splenic function. Eur. J. Clin. Microbiol. Infect. Dis..

[54] Mittal S., Ali J., Baboota S. (2021). Enhanced anti-psoriatic activity of tacrolimus loaded nanoemulsion gel via omega 3-Fatty acid (EPA and DHA) rich oils-fish oil and linseed oil. J. Drug Deliv. Sci. Technol..

[55] Van der Fits L., Mourits S., Voerman J.S., Kant M., Boon L., Laman J.D., Cornelissen F., Mus A.-M., Florencia E., Prens E.P. (2009). Imiquimod-induced psoriasis-like skin inflammation in mice is mediated via the IL-23/IL-17 axis. J. Immunol..

[56] Zambaux M.F., Bonneaux F., Gref R., Maincent P., Dellacherie E., Alonso M., Labrude P., Vigneron C. (1998). Influence of experimental parameters on the characteristics of poly (lactic acid) nanoparticles prepared by a double emulsion method. J. Control. Release.

[57] Torchilin V.P. (2014). Multifunctional, stimuli-sensitive nanoparticulate systems for drug delivery. Nat. Rev. Drug Discov..

[58] Talib S., Ahmed N., Khan D., Khan G.M., Rehman A.u. (2021). Chitosan-chondroitin based artemether loaded nanoparticles for transdermal drug delivery system. J. Drug Deliv. Sci. Technol..

[59] Mohammed A.M., Osman S.K., Saleh K.I., Samy A.M. (2020). In vitro release of 5-fluorouracil and methotrexate from different thermosensitive chitosan hydrogel systems. AAPS PharmSciTech.

[60] Owen D.H., Peters J.J., Katz D.F. (2000). Rheological properties of contraceptive gels. Contraception.

[61] Dantas M.G.B., Reis S.A.G.B., Damasceno C.M.D., Rolim L.A., Rolim-Neto P.J., Carvalho F.O., Quintans-Junior L.J., Almeida J.R.G.d.S. (2016). Development and evaluation of stability of a gel formulation containing the monoterpene borneol. Sci. World J..

[62] Khalil R.M., Abd El-Bary A., Kassem M.A., Ghorab M.M., Ahmed M.B. (2013). Solid lipid nanoparticles for topical delivery of meloxicam: Development and in vitro characterization. Eur. Sci. J..

[63] Ogata A., Kumanogoh A., Tanaka T. (2012). Pathological role of interleukin-6 in psoriatic arthritis. Arthritis.

[64] Bajaj S., Singla D., Sakhuja N. (2012). Stability testing of pharmaceutical products. J. Appl. Pharm. Sci..

[65] Guideline I.H.T. (2003). Stability testing of new drug substances and products. Q1A (R2) Curr. Step.

[66] Luo Y., Wang Q. (2014). Recent development of chitosan-based polyelectrolyte complexes with natural polysaccharides for drug delivery. Int. J. Biol. Macromol..

[67] Van Gheluwe L., Chourpa I., Gaigne C., Munnier E. (2021). Polymer-based smart drug delivery systems for skin application and demonstration of stimuli-responsiveness. Polymers.

[68] Klee S.K., Farwick M., Lersch P. (2009). Triggered release of sensitive active ingredients upon response to the skin’s natural pH. Colloids Surf. A Physicochem. Eng. Asp..

[69] Hari B.V., Narayanan N., Dhevendaran K., Ramyadevi D. (2016). Engineered nanoparticles of Efavirenz using methacrylate co-polymer (Eudragit-E100) and its biological effects in-vivo. Mater. Sci. Eng. C.

[70] Danaei M., Dehghankhold M., Ataei S., Hasanzadeh Davarani F., Javanmard R., Dokhani A., Khorasani S., Mozafari M. (2018). Impact of particle size and polydispersity index on the clinical applications of lipidic nanocarrier systems. Pharmaceutics.

[71] Turner P.V., Brabb T., Pekow C., Vasbinder M.A. (2011). Administration of substances to laboratory animals: Routes of administration and factors to consider. J. Am. Assoc. Lab. Anim. Sci..

[72] Singhvi G., Singh M. (2011). In-vitro drug release characterization models. Int. J. Pharm. Stud. Res..

[73] Pischon H., Radbruch M., Ostrowski A., Volz P., Gerecke C., Unbehauen M., Hönzke S., Hedtrich S., Fluhr J.W., Haag R. (2017). Stratum corneum targeting by dendritic core-multishell-nanocarriers in a mouse model of psoriasis. Nanomed. Nanotechnol. Biol. Med..

