# Supplementary material for: Development and In Vitro/In Vivo Evaluation of pH-Sensitive Polymeric Nanoparticles Loaded Hydrogel for the Management of Psoriasis
Source: Nanomaterials (Basel). 2021 Dec 17;11(12):3433. doi: 10.3390/nano11123433 (PMC8705938; doi:10.3390/nano11123433)
Supplement: Supplementary file 1 [file nanomaterials-11-03433-s001.zip › nanomaterials-1492650-Supplementary Materials.pdf]

Supplementary Materials

Development and In Vitro/In Vivo Evaluation of pH-Sensitive Polymeric Nanoparticles Loaded Hydrogel for the Management of Psoriasis

Muhammad Imran Asad [1], Dildar Khan [1], Asim ur Rehman [1], Abdelhamid Elaissari [2] and Naveed Ahmed [1,*]

[1] Department of Pharmacy, Quaid-i-Azam University, Islamabad 45320, Pakistan; miasad@bs.qau.edu.pk (M.I.A.); dildarafridi3@gmail.com (D.K.); arehman@qau.edu.pk (A.u.R.)
[2] CNRS, Univ Lyon, University Claude Bernard Lyon–1, ISA-UMR 5280, 69622 Villeurbanne, France; elaissari@lagep.univ-lyon1.fr
* Correspondence: author: natanoli@qau.edu.pk

Table S1. Kinetics models for drug release from NPs and hydrogel

	R^2 Value for MTX NPs				
**Type of formulation**	**Zero Order**	**First Order**	**Higuchi Model**	**Korsmeyer-Pappas**	**Hixson-Crowell**
**MTX loaded NPs (pH 5)**	0.9433	0.9901	**0.9944**	0.9535	0.9788
**MTX loaded NPs (pH 7.4)**	0.8731	0.8834	**0.9952**	0.9056	0.8788
**Type of formulation**	R^2 Value for MTX NPs loaded hydrogel				
	**Zero Order**	**First Order**	**Higuchi Model**	**Korsmeyer-Pappas**	**Hixson-Crowell**
**MTX loaded NPs hydrogel (pH 5)**	0.9048	0.9559	**0.999**	0.9419	0.9407
**MTX loaded NPs hydrogel (pH 7.4)**	0.9034	0.9116	**0.9984**	0.9357	0.9077
